# New Molecular Data on Filaria and its *Wolbachia* from Red Howler Monkeys (*Alouatta macconnelli*) in French Guiana—A Preliminary Study

**DOI:** 10.3390/pathogens9080626

**Published:** 2020-07-31

**Authors:** Younes Laidoudi, Hacène Medkour, Anthony Levasseur, Bernard Davoust, Oleg Mediannikov

**Affiliations:** 1 IRD, AP-HM, Microbes, Evolution, Phylogeny and Infection (MEPHI), IHU Méditerranée Infection, 19–21, Bd Jean Moulin, 13005 Marseille, France; younes.laidoudi@yahoo.com (Y.L.); hacenevet1990@yahoo.fr (H.M.); anthony.levasseur@univ-amu.fr (A.L.); bernard.davoust@gmail.com (B.D.); 2 Aix Marseille Univ, 19–21, Bd Jean Moulin, 13005 Marseille, France

**Keywords:** *Mansonella* sp., *Brugia* sp., Onchocercidae sp., *Wolbachia*, neotropic monkeys, reservoir, zoonosis

## Abstract

Previous studies have reported filarial parasites of the genus *Dipetalonema* and *Mansonella* from French Guiana monkeys, based on morphological taxonomy. In this study, we screened blood samples from nine howler monkeys (*Alouatta macconnelli*) for the presence of filaria and *Wolbachia* DNA. The infection rates were 88.9% for filaria and 55.6% for wolbachiae. The molecular characterization, based on the *18S* gene of filariids, revealed that *A. macconnelli* are infected with at least three species (*Mansonella* sp., *Brugia* sp. and an unidentified Onchocercidae species.). Since the *18S* and *cox1* generic primers are not very effective at resolving co-infections, we developed ITS genus-specific PCRs for *Mansonella* and *Brugia* genus. The results revealed coinfections in 75% of positives. The presence of *Mansonella* sp. and *Brugia* sp. was also confirmed by the *16S* phylogenetic analysis of their associated *Wolbachia*. *Mansonella* sp., which close to the species from the subgenus *Tetrapetalonema* encountered in New World Monkeys, while *Brugia* sp. was identical to the strain circulating in French Guiana dogs. We propose a novel *ITS1* *Brugia* genus-specific qPCR. We applied it to screen for *Brugia* infection in howler monkeys and 66.7% were found to be positive. Our finding highlights the need for further studies to clarify the species diversity of neotropics monkeys by combining molecular and morphological features. The novel *Brugia* genus-specific qPCR assays could be an effective tool for the surveillance and characterization of this potential zoonosis.

## 1. Introduction

Filariasis unites diseases are caused by arthropod-borne filariids and nematodes belonging to the Onchocercidae family. Several species can be encountered in human and animals with some zoonotic aspects. Morphologically, the adult filariids are long, string-like, white-to-cream-colored worms [1]. They appear to be capable of living inside various tissues and cavities outside the gastrointestinal tract. Once mature, the adult females produce blood or cutaneous microfilariae, where they are available to arthropod vectors [2]. Species having a predilection for subcutaneous tissues are less or completely avirulent in comparison to those found in cavities, such as *Dipetalonema* species (*D. gracile, D. graciliformis, D. caudispina, D. robini* and *D. freitasi, D. vanhoofi*), *Macacanema formosana* where they induce serious disease manifestations such as pleuritis, fibrinopurulent peritonitis and fibrinous adhesion, resulting in the entrapment of worms [3,4]. Furthermore, species found in the circulatory system (e.g., *Dirofilaria immitis* and *D. pongoi, Edesonfilaria malayensis*), as well as those present in the lymphatic system, such as Brugian filariids (*B. malayi, B. pahangi, B. timori* and *B. tupaiae*) and *Wuchereria bancrofti,* disrupt blood and lymphatic drainage, leading to serious and often irreversible vascular damage [4,5,6,7,8,9]. These filariids, along with *Onchocerca volvulus*, the agent of river blindness, constitute the most thread-like filarial worms and have affected up to 893 million people in 49 countries worldwide [10].

Several filariids of the subfamilies Onchocercinae and Dirofilariinae are associated with an endosymbiotic intracellular bacterium of the genus *Wolbachia* [11], which is present in all developmental stages of filariids that harbor *Wolbachia*, leading to their long-term survival [12]. The parasites’ endosymbiotic *Wolbachia* are implicated in severe inflammatory-mediated filarial diseases [13,14,15,16]. Anti-wolbachial therapies, based on the administration of antibiotics, are known to be effective against the most common filariasis caused by *Brugia* spp., i.e., *W. bancrofti, Mansonella perstans* and *D. immitis* [17,18,19]. The *Wolbachia*-filaria relationship is species-specific, wherein each filariid has a specific genotype of *Wolbachia* [11], thus providing an additional target suitable for the diagnosis of filarial infections [20], especially when occurring in dead-end hosts, as is the case in *D. immitis* in human and cats [21,22]. Recently, the simultaneous detection of both filarial and wolbachial DNAs from infected hosts is used as an improvement tool for the diagnosis of filarial infections [23,24,25].

Filariasis is one of the most neglected tropical diseases selected, but it is included in the Mass Drug Administration (MDA) program to achieve its elimination by 2020 [26,27,28]. Human filariasis was almost eliminated from Latin America [29,30]. Thanks to the MDA program, river blindness (onchocerciasis caused by *O. volvulus*) transmission is currently limited to the Amazon rainforest on the Venezuelan–Brazilian border, while the lymphatic filariasis caused by *W. bancrofti* only occurs in four countries: Brazil, the Dominican Republic, Guyana, and Haiti [31]. Another human sympatric filariasis caused by *M. ozzardi* and *M. perstans* occurs today in a small foci in South America (Amazon Basin, Yucatan, Panama and Haiti) [32,33,34]. In Latin America, domestic and wild animals seem to be the foci of some neglected filariasis potentially zoonotic such as *Brugia guyanensis* (Orihel 1964) from the lymphatic system of the coatimundi (*Nasua nasua vittata*) in French Guiana [35] and some unidentified Brugian filariids in dogs and ring-tailed coatis (*Nasua nasua*) [25,36], and the zoonotic canine filariasis (e.g., *D. immitis* and *Acanthocheilonema reconditum*) from Brazil and French Guiana [25,37].

New world monkeys are a diverse group of arboreal primates inhabiting the tropical forest environments of southern Mexico, Central and South America [38]. These primates are the natural hosts for several filariids belonging to the genus *Dipetalonema* and *Mansonella*, where they are often present as co-infected [3,39]. Howler, monkeys (*Alouatta* spp., Atelidae, Primata) have a wide distribution, from Mexico to northern Argentina. Only a few species of this group have been genetically characterized [40]. The red howler monkey (*Alouatta macconnelli*, Linnaeus 1766—Elliot 1910) is one of eight species of primates found in the French Guiana forest [41]. They are medium sized (10 kg) and about 84 cm (head and body) with a prehensile tail [38]. They live in small groups of four to eight individuals. The primary forest in the canopy high strata is often frequented by these primates who are mainly found in the north of South America and the Amazonia (Suriname, Guyana, Trinidad, French Guiana, Venezuela and Brazil). Their diet is low in energy (leaves and sometimes fruits and seeds) [40]. Population density is estimated to be 13 individuals/km^2^ along the Approuague River, which is the location in which we conducted our investigation [42]. Nowadays, little molecular data are available on filarial parasites in howler monkeys from French Guiana. The aims of the present study are mainly to determine, at the molecular level, the presence of filarial parasites and the status of their endosymbiotic *Wolbachia* in red howler monkeys. To this end, we examined blood samples obtained from a game that was hunted by the natives of French Guiana [43].

## 2. Results

### 2.1. Host Identification

**Figure 1:**
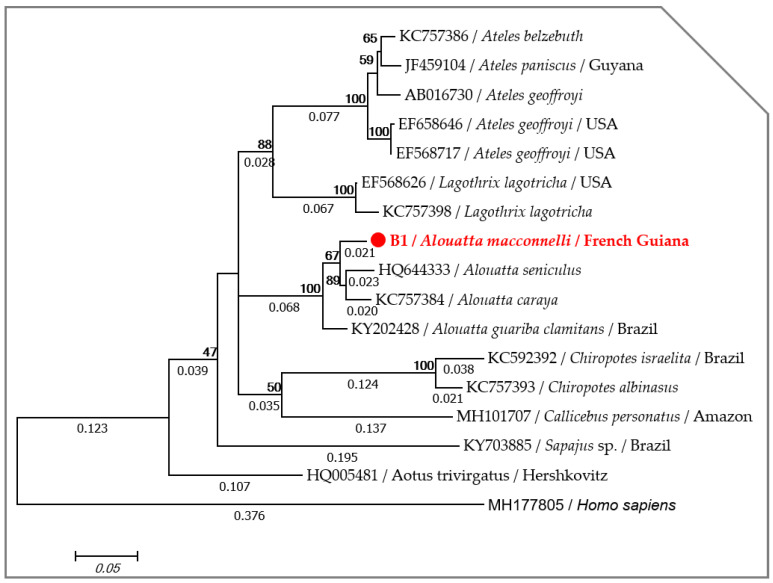
Phylogram generated by maximum likelihood method from 17 partial (521 bp) *cox1* sequences showing the position of *Alouatta macconnelli* through the neotropics monkeys. A discrete Gamma distribution was used to model evolutionary rate differences among the sites (5 categories (*+G*, parameter = 0.4575)). The rate variation model allowed for some sites to be evolutionarily invariable ([*+I*], 57.2649% sites). Likelihood was −2676.5239. Numbers above and below the branches display the nod statistics and branch length, respectively. Geographical location (when available) and GenBank accession numbers are indicated in each node.

Folmer’s primers allowed for the amplification of DNA sequences from all blood samples, but despite several attempts, a high-quality DNA sequence of the vertebrate *cox1* gene was only obtained in one from among the nine samples tested, suggesting the presence of a non-specific amplification from the latter. The partial nucleotide sequence (558 bp) of the *cox1* gene obtained in this study was deposited in the GenBank under accession number MT193011. Blast analysis showed that the *cox1* sequence of howler monkeys in our study had an identity of 96.06% with *Alouatta seniculus* (HQ644333), 95.88% with *Alouatta caraya* (KC757384) and 95.34% with *Alouatta guariba* (KY202428) and a query cover of 100%. Accordingly, the phylogenetic analysis using the Maximum Likelihood (ML) method showed that the specimen of howler monkeys (*Alouatta macconnelli*) is monophyletic with other *Alouatta* species (Figure 1).

### 2.2. Molecular Screening for Filarial and Wolbachia DNAs in Howler Monkeys

Filarial and *Wolbachia* DNAs were detected by qPCR assays in eight out of nine samples tested and six out of nine samples tested, which correspond to a frequency of infection of 88.9% and 66.7% for filaria and *Wolbachia,* respectively. This is the first molecular report of filaria and its *Wolbachia* from the howler monkeys of French Guiana.

### 2.3. Molecular Characterization of Filarial Species

To identify filaria detected by qPCR. we performed standard polymerase chain reaction (PCR) screening with primers targeting the small subunit rRNA (*18S)*, the internal transcribed spacer 1 (*ITS1*) and the cytochrome c oxidase subunit I (*cox1*) genes. A nearly full-length DNA sequence of the *18S* rRNA gene was obtained from all eight samples, was positive in qPCR and was split into three isolates according to the blast results. (i) Six sequences were obtained from the monkeys B2, B3, B4, B6, B7 and B8. These amplicon sequences were identical to each other, showing an identity and query cover of 100% with *Dipetalonema* sp. (DQ531723) isolated from an owl monkey (*Aotus nancymaae*) captured in Peru and 99.6% of identification with the *Mansonella* species (MN432520, MN432519). (ii) One *18S* sequence obtained from sample B5 was very close to the Onchocercidae members (*Onchocerca cervicalis:* DQ094174, and *Loa loa*: DQ094173), where the identification was 99.9% and 100% of the query cover. Further sequence comparisons showed that the Adenine and Thymine mutated into Cytosine at the position 304 and 879 with *O. cervicalis* (DQ094174) and *L. loa* (DQ094173), respectively (Figure S1). (iii) One sequence from sample B9 showed an identification of 100% with *B. malayi* (AF036588) and 99.9% with *Brugia* sp. (MN795087), isolated from dogs in French Guiana.

*Mansonella* genus-specific PCR, based on the amplification of the *ITS1*, allowed us to obtain ITS sequences of *Mansonella* sp. from seven monkeys (B2, B3, B4, B5, B6, B7 and B8). They were almost identical and displayed an identity ranging from 83.47% to 93.49% and a query cover ranging from 62% to 83% with *Mansonella* species (*M. ozzardi*: KR952332, *M. perstans*: MN432520, *M. mariae*: AB362562, *M. streptocerca*: KR868771, *M. dunni*: KY434312 and *Mansonella* sp.: MN821052). Furthermore, *Brugia* sp. was identified in five samples (B2, 3, 4, 7 and 9) using the *Brugia*-specific qPCR and ITS sequences were obtained for four of them. These sequences were similar and were close to the *Brugia* species, wherein the identity ranged from 88.81% to 91.98% with *B. malayi* (JQ327147, EU419333) and from 89.10% to 91.19% with *B. pahangi* (EU373633, EU419348).

Primers targeting the *cox1* gene amplified the expected DNA amplicon size from all the filaria-positive samples. However, only two sample (B8 and B9) sequences provided good quality electropherograms. Several overlapping peaks (double peaks) within samples B2, B3, B4, B5, B6 and B7 suggested co-infection with two or more filarial species. Blast analysis showed that the specimen amplified from monkey B8 had an identity of 88.2% with *Mansonella perstans* (MN890111). While the *cox1* sequence amplified from monkey B9 was very close to Brugian filariids, with an identity of 99.6% with *Brugia* sp. (MT193074), isolated from dogs in French Guiana, 95.4% with *Brugia timori* (AP017686) and 94.9% with *Brugia malayi* (MN564741).

**Figure 2:**
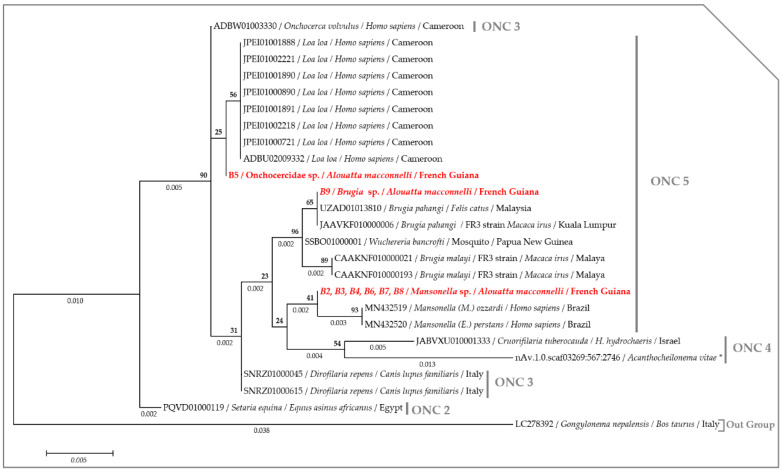
Phylogram generated by Maximum Likelihood (ML) method based on 24 partial (941 bps) rRNA sequences showing the position of filariids from howler monkeys Onchocercidae clades (ONC). A discrete Gamma distribution was used to model evolutionary rate differences among the sites (5 categories (*+G*, parameter = 0.1000)). The likelihood was −1770.1752. Numbers above and below the branches display nod statistics and branch lengths, respectively. Geographical location (when available) and GenBank accession numbers are indicated in each node. (*) indicates sequences retrieved from the Worm parasites database.

Phylogenetic analysis using the maximum likelihood method of the *18S* rRNA gene showed that howler monkeys from French Guiana are infected with at least three filarial species belonging to the Onchocercidae clade, namely ONC 5. The *18S* sequences amplified from monkeys B2, 3, 4, 6, 7 and 8 clustered in a separate branch with *Mansonella* species, while the sequence obtained from monkey B5 appeared paraphyletic with respect to *L. loa* (ADBU02009332) and *O. volvulus* (ADBW01003330), suggesting an unknown onchocercid. Finally, the sequence from monkey B5 clustered with the *B. pahangi* strain (UZAD01013810 and JAAVKF010000006) (Figure 2).

**Figure 3:**
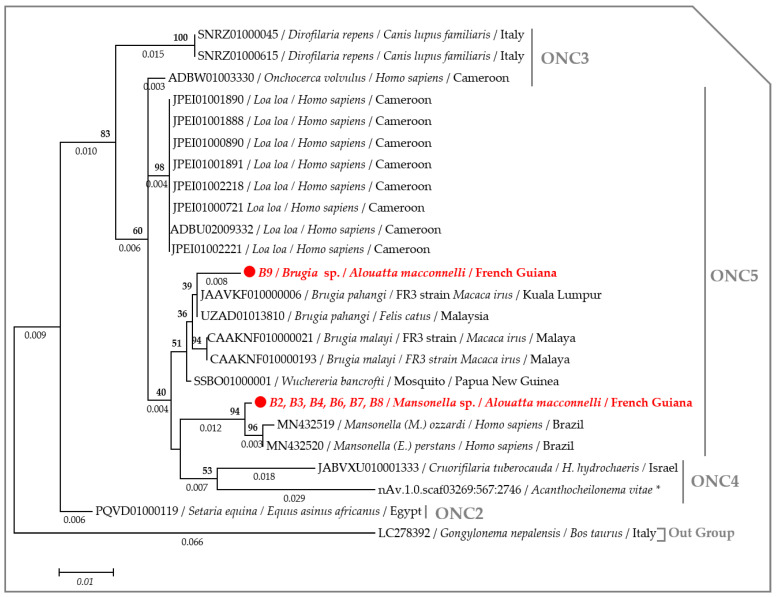
Phylogram generated by ML method based on 24 partitioned concatenated rRNA sequences (*18S* ad *ITS1*) showing the position of *Brugia* sp. and *Mansonella* sp. through Onchocercidae clades (ONC). The total length was 1221 bp, the rate variation model allowed for some sites to be evolutionarily invariable ([*+I*], 29.0648% sites). Likelihood was −3034.4989. Numbers above and below the branches display nod statistics and branch lengths, respectively. Geographical location (when available) and GenBank accession numbers are indicated in each node. (*) indicates sequences retrieved from Worm parasites database.

**Figure 4:**
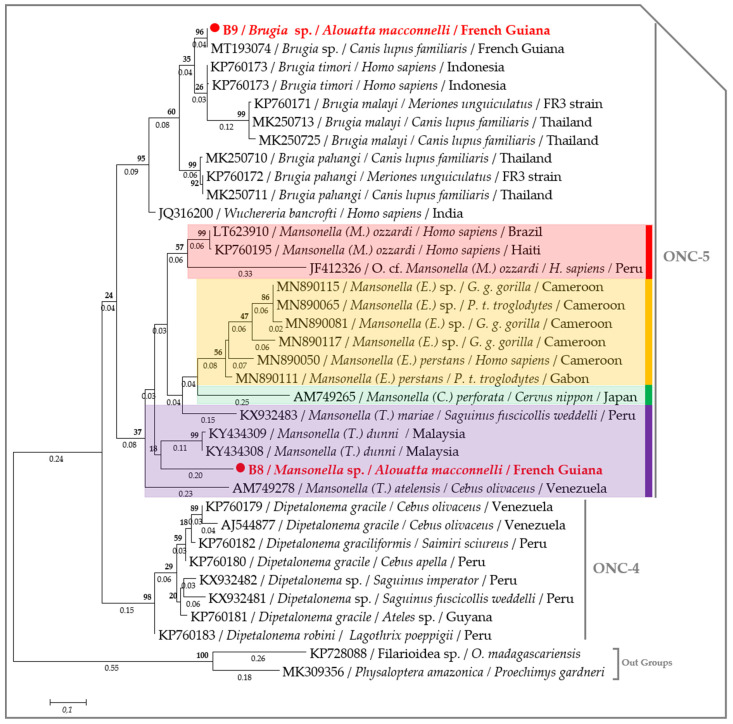
Phylogram generated by ML method based on 36 *cox1* partial sequences (266 bp) showing the position of *Brugia* sp. and *Mansonella* sp. through Onchocercidae clades (ONC). A discrete Gamma distribution was used to model evolutionary rate differences among the sites (five categories (*+G*, parameter = 0.4964)). The rate variation model allowed for some sites to be evolutionarily invariable ([*+I*], 0.000% sites). The likelihood was −2194.0587. Numbers above and below the branches display nod statistics and branch lengths, respectively. Host, geographical location (when available) and GenBank accession numbers are indicated in each node. *Mansonella* species are color-coded according to their subgenus.

**Figure 5:**
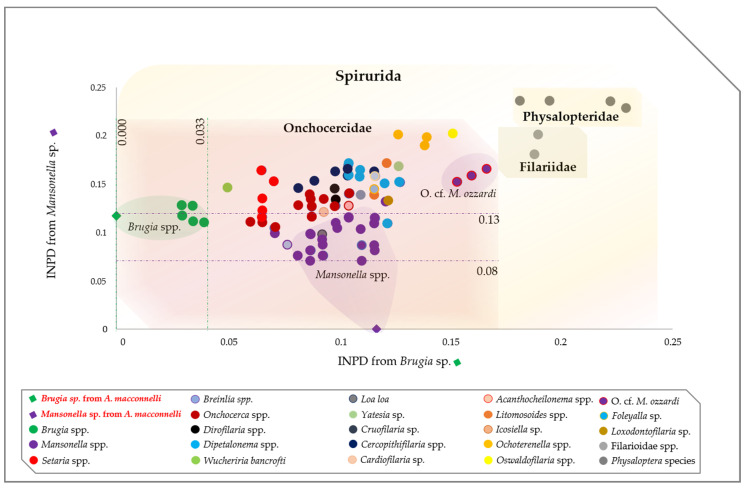
Scatter chart showing the interspecific pairwise distance between the cox1 sequences of *Brugia* sp. (abscissa) and *Mansonella* sp. (ordinate) from *A. macconnellii* and the representative members of Onchocercidae clades. The analyses involved 112 partial (266 bp) cox1 sequences with a total of 216 positions in the final dataset. All positions containing gaps and missing data were eliminated.

The ML tree, based on the concatenated rRNA sequences (*18S* and *ITS1*), showed that the specimens amplified from monkeys B2, 3, 4, 6, 7 and 8 clustered with other monophyletic species of the genus *Mansonella*, while the specimen amplified from monkey B9 clustered with the *Brugia* species (Figure 3). Interestingly, the *cox1* phylogram replicated the same results, though with a greater degree of accuracy. The species amplified in this study belong to the clade 5 of the Onchocercidae family. More precisely, the species amplified from monkey B8 belong to the genus *Mansonella* and the subgenus *Tetrapetalonema* encountered in New World Primates [44], while the species from monkey B9 clustered with *Brugia* sp. (MT193074), isolated from dogs in French Guiana [45] and are monophyletic with other Brugian filariids (Figure 4). Interspecific nucleotide distances (IND) of the *cox1* sequences ranged between 0.08 and 0.13 between *Mansonella* sp. from the monkey B8 and most species from the genus *Mansonella* (MN890075, MN890115, MN890111 and KY434309), while the IND ranged from 0 to 0.03 between *Brugia* sp. amplified from monkey B9 and Brugian filariids (Figure 5, Table S1).

**Figure 6:**
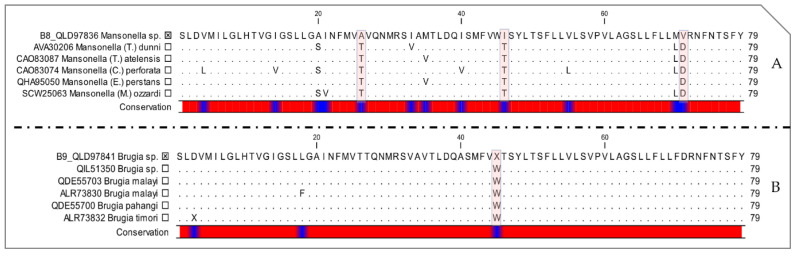
Cytochrome c oxidase subunit I protein sequences (COI) alignment showing the conservation of amino acid within (**A**) *Mansonella* spp., (**B**) *Brugia* spp. Protein Id and species name are indicated for each sequence. Selected boxes represent species obtained in this study.

Importantly, the *cox1* DNA sequences were aligned correctly to the reference mitogenome of *M. ozzardi* (KX822021) [45], and when translated, there were no stop codons in the amino acid sequences, suggesting the absence of co-amplified numts. Finally, translated protein sequences of the cytochrome c oxidase subunit I (COI) showed three amino acid changes between *Mansonella* sp. from monkey B8 and the other *Mansonella* species from GenBank, namely, from threonine to alanine, threonine to isoleucine and aspartic acid to valine (Figure 6A). While *Brugia* sp. from monkey B9 showed a deletion of one amino acid instead of tryptophan, in comparison to Brugian filariids from GenBank (Figure 6B).

**Figure 7:**
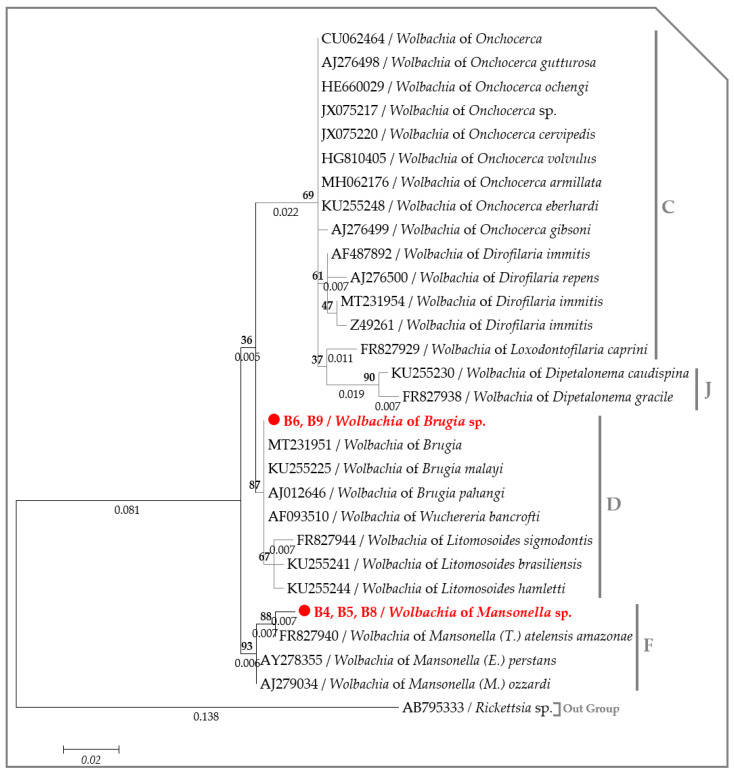
Phylogram generated by the maximum likelihood method based on 29 nucleotide sequences of the partial (295 bp) *16S* gene showing the position of *Wolbachia* of *Brugia* sp. and *Mansonella* sp. through *Wolbachia* of filarial nematodes. The likelihood was −777.8125. A discrete Gamma distribution was used to model evolutionary rate differences among the sites (5 categories (*+G*, parameter = 0.2802)). Numbers above and below the branches display nod statistics and branch lengths, respectively. Filarial host and GenBank accession numbers are indicated in each node.

A partial DNA sequence of the *Wolbachia 16S* gene (295 bps) was obtained from five out of six samples that tested positive for *Wolbachia* DNA through the qPCR. Three identical sequences revealed 99.32% identity with *Wolbachia* of *M. atelensis amazonae* (FR827940) and 98.64% with both *Wolbachia* of *M. perstans* (AY278355) and *M. ozzardi* (AJ279034). These sequences were obtained from filaria-positive monkeys, including monkey B4, which was co-infected with *Mansonella* sp. and *Brugia* sp., monkey B5 co-infected with an unidentified Onchocercidae species and *Mansonella* sp. and monkey B8, which was mono-infected with *Mansonella* sp. The two remaining sequences were amplified from two filaria-positive samples, one for *Mansonella* sp. (B6) and the other for *Brugia* sp. (B9). These sequences were identical with each other and were 100% identical with all *Wolbachia* genotypes associated to *Brugia* species (CP050521, CP034333, AJ012646 and MT231956). Accordingly, the ML inference indicates that the *Wolbachia* genotype from monkeys B4, 5 and 8 belong to the Clade F of *Wolbachia* lineage infecting *Mansonella* species, while the genotype obtained from monkeys B6 and B9 clustered together with *Wolbachia* endosymbiont of Brugian filariids within Clade D of the *Wolbachia* lineage (Figure 7).

Finally, by combining all the molecular results for filaria and *Wolbachia* detection, we concluded six cases (75%) of co-infections in monkeys, including *Mansonella* sp.—*Brugia* sp. co-infection in five and *Mansonella* sp.—unidentified Onchocercidae species in one. Two other monkeys (25%) presented mono-infections, one with *Mansonella* sp. and the other with *Brugia* sp. (Table 1).

## 3. Discussion

This is the first molecular report of filaria and *Wolbachia* infection from red howler monkeys (*Alouatta macconnelli*, Linnaeus 1766—Elliot 1910) in French Guiana. These monkeys were morphologically considered as a distinct species from *A. seniculus* and they are not a subspecies [46]. Our data confirmed that, molecularly, both species can be distinguished by their *cox1* sequences. The wide distribution of howler monkeys (from Mexico to northern Argentina) constitutes a non-negligible reservoir for zoonotic disease [43] and should be monitored. Our study is limited in the number of species and samples, due to the difficulties encountered in the field. The number of monkeys tested was much lower than those tested in Reference [47], where 1353 free-ranging mammals, including 114 howler monkeys (*A. seniculus*) and 84 red handed tamarins (*Saguinus midas*) from the neotropical primary rainforest in French Guiana were studied for haemoparasites and microfilariae. However, the prevalence of filarial infection we recorded using molecular assays is close to that reported in tamarins and howler monkeys using blood smear, where the infection rates were 80% and 92% of filaria infections (*Dipetalonema* and *Mansonella* (*Tetrapetalonema*) species), respectively [47]. Our data indicate that the prevalence of filarial infection was higher than that of sloths, anteaters and porcupines in French Guiana, where the infection rate of 40% was reported using blood smears test [47]. The higher prevalence observed in monkeys may be related to the lower host specificity of filariids [48] and/or similar biotope of potential vectors [49]. Another hypothesis is that the lifestyle of these animals increases the risk of vector-borne disease transmission between infected and non-infected individuals in the monkey colony. Therefore, the highest mixed-infection detected in our study corroborates previous reports [50], but it is still unknown whether it is geographical or host-specific. Several species of filariids are reported from a wide range of neo-tropical primates based on morphological taxonomy (Table 2). Most of them belong to the genus *Dipetalonema* and *Mansonella* (*Tetrapetalonema*). However, data in DNA barcoding of these species is lacking.

The use of two (or more) different molecular markers for species delimitation remained necessary for the accurate identification of nematode species [51]. In the present study, our molecular approach, based on generic and genus specific primers, permits the detection and characterization of filarial infections and resolved the co-infections. This is due to the ability of ITS genus-specific PCR assays to separately amplify DNA amplicons depending on their specificity. Filarial nematodes could be misclassified when the *18S* gene is used alone as a barcode. This gene is often limited to the genus level and has proven to be inconclusive for the molecular taxonomy of nematodes [52], while the ITS 1 gene appears to be a satisfactory barcode in resolving taxonomic relationships among species [53,54,55]. Furthermore, as suggested by previous authors [56], the use of partitioned concatenated DNA sequences enables the accurate identification of filarial nematodes. We used both the *18S* and the partitioned concatenated rRNA (*18S* and *ITS1*) gene, which confirmed the presence of at least three potential new species from clade 5 of the Onchocercidae family present in howler monkeys in French Guiana, including *Mansonella* sp., *Brugia* sp. and an unidentified Onchocercidae species.

The *cox1* gene enabled the accurate identification of the *Mansonella* species from wild non-human primates from Cameroon and Gabon [57], and has been proven to be a satisfactory discrimination between filarial species. This gene was described by its low nucleotide distances (from 0 to 0.02) within filarial species [58] and a larger variation between congeneric species (i.e., 0.098 to 0.2) [58,59]. In the present study, we used two different phylogenetic methods for the analysis of *cox1*, together with the alignment of COI protein sequences, which confirmed that species from monkeys B8 and B9 clustered, respectively, with *Mansonella Tetrapetalonema* subgenus and *Brugia* species, with the distance ranging between 0.02 and 0.2, suggesting unidentified or potential new species from these genera.

*Wolbachia* are host-specific, and each genotype is associated with a specific filarial species [11,60]. Bacterial genotype-specific identification was previously proposed for the speciation of *Brugia* parasites that infect humans [9]. Several studies showed the utility of the specific detection of *Wolbachia* in determining the subject as infected or not with filarial species (e.g., *D. immitis, D. repens, B. pahangi* and *B. malayi*) from domestic animals [14,21,23,24,25,61,62]. Accordingly, the phylogenetic analysis of the *Wolbachia 16S* DNA sequences demonstrated the presence of two bacterial genotypes belonging to the supergroup F and D encountered in *Mansonella* and *Brugia* species, thus corroborating with filaria phylogenies. The inconsistency between the bacterial genotype and filaria species was observed in monkey B6. The presence of *Mansonella* sp. and *Wolbachia* of *Brugia* sp. DNAs highlights a co-infection with both filarial species. However, the absence of *Wallachia* of *Mansonella* sp. could be explained by a weaker infection density in this species, while the absence of *Brugia* sp. DNA, despite the presence of its *Wolbachia,* could be result to an amicrofilaremic infection due to single sex infection, an earlier infection stage or any other causes. Such inconsistencies were previously reported between *Brugia* and *Dirofilaria* species in dogs [63]. *Wolbachia*-filaria interactions within co-infected hosts are not well understood. Despite the presence of both parasites in co-infected dogs with *D. immitis* and *D. repens,* the single detection of *Wolbachia* of *D. immitis* is frequent [24] and may result in an unexplained suppression effect on the production of *D. immitis* microfilariae induced by the presence of *D. repens* [64,65].

Our findings extend the presence of *Brugia* sp. and an unidentified Onchocercidae species to the New World Monkeys (e.g., *Alouatta macconnelli*). Several species of filariae have been described from these primates and they all belong to the genus *Dipetalonema* or *Mansonella* subgenus *Tetrapetalonema* [4] (Table 2). The genus *Dipetalonema* (Diesing 1861) is restricted to non-human primates (NHPs) of the neotropics, according to the phylogenetic study conducted by Lefoulon et al. [56]. Adult worms are prevalent in the serous cavities of the hosts. A high species diversity of this genus was observed in a wide range of New World monkeys. *D. gracile* (Rudolphi 1819), *D. graciliformis* (Freitas 1964) and *D. caudispina* (Molin 1858) are the main species found in Guiana monkeys, using a morphological taxonomy (Table 2).

The subgenus *Mansonella* (*Tetrapetalonema*) is one of the five subgenera derived from the genus *Mansonella*. Adult filariids are small, slender and can be found in subcutaneous tissues. The *Tetrapetalonema* subgenus comprises 13 species (Table 2), which have been restricted to platyrrhine (neotropical) primates [66]. Human mansonellensiasis across South America regions are caused by *M. ozzardi* type species of *Mansonella* (*Mansonella*) subgen. n. [44,45] causing fever, pruritis, arthralgias, headache, rashes, lymphadenopathy, edema, and pulmonary symptoms and a common eosinophilia mainly associated with corneal lesions [67,68,69,70]. *M. perstans* type species of *Mansonella* (*Esslingeria,* Chabaud and Bain 1976) subgen. n. [44] is another agent of human mansonellensiasis in some neotropical regions of Central and South America that causes the bung-eye diseases [71]. These species have been found in both humans and non-human primates [4,44]. However, the possibility that the *Mansonella* sp. we have detected here is one of the 13 *Mansonella* (*Tetrapetalonema*) species or a new species from this subgenus cannot be ruled out in the absence of morphological identification.

*Brugia* spp. are incidental filariids that parasitize non-human vertebrates [72]. The classical brugian filariids involved in lymphatic filariasis are found in Asia, while species reported from North and South America constitute the most zoonotic species of this genus [73]. In Latin America, *Brugia* sp. infection was reported from the ring-tailed coatis (*Nasua nasua nasua*) in Brazil [36], *Brugia guyanensis* from the lymphatic system of the coatimundi (*Nasua nasua vittata*) in British Guiana [35] and *Brugia* sp. from domestic dogs in French Guiana [25]. Our findings indicate that *Brugia* sp. detected from howler monkeys is the same as that recently detected in domestic dogs [25]. Unlike Asian primates in which infection with *B. malayi* and *B. pahangi* has been reported [74], Brugian filariid has not been reported in neotropical primates [75]. Cases of human infection by *Brugia* sp. have been reported in several localities (Amazon, Peru, Colombia) in South America, but the reservoir of the parasites is unknown [72,73]. However, the possibility that the *Brugia* sp. we detected from howler monkeys and dogs in our previous study [25] is of the same species circulating in humans cannot be ruled out in the absence of molecular data.

## 4. Materials and Methods

### 4.1. Samples and Ethic Statement

In January 2016, we obtained samples from howler monkeys that were legally hunted by two Amerindian hunters for family consumption of meat. The International Union for Conservation of Nature conservation status for this species is a “least concern” [83,84]. The hunters applied the provisions of the prefectural decree regulating the quotas of species that can be taken by a person in the department of Guiana (No. 583/DEAL of 12 April 2011). The hunt took place in the deep forest (4°01′39.5″ N 52°31′32.5″ W), near the Approuague River, 50 km from the village of Regina. We were able to examine corpses of nine hunted howler monkeys (five females and four males). Blood was collected by a heart-puncture in sterile tubes containing Ethylene-Diamine-Tetra-Acetic acid (EDTA) and was kept in a cooler before being frozen at −20 °C until further analysis.

### 4.2. DNA Extraction

Genomic DNA was extracted from 200 µL of each blood samples. The extraction was performed using QIAGEN DNA tissues kit (QIAGEN, Hilden, Germany) following the manufacturer’s recommendations. Two lysis steps were applied before the extraction procedure: (i) mechanical lyses performed on FastPrep-24™ 5G homogenizer using high speed stirring for 40 s in the presence of glass powder, (ii) enzymatic digestion of proteins with buffer G2 and proteinase K for 12 h at 56 °C. The extracted DNA was eluted in a total volume of 100 µL and stored at −20 °C.

### 4.3. Host Identification

The universal *cox1* DNA barcoding region of metazoans [85] was targeted using the degenerated primers of Folmer, as described elsewhere [86]. The PCR products were purified, sequenced and edited, as described below, and were then aligned against *cox1* sequences of *Alouatta* spp. (HQ644333, KC757384, KY202428), *Ateles* spp. (AB016730, KC757386, JF459104, EF658646, EF568717), *Callicebus personatus* (MH101707), *Chiropotes israelita* (KC592392, KC757393), *Lagothrix lagotricha* (EF568626, KC757398), *Sapajus* spp. (KY703885) and *Aotus trivirgatus* (HQ005481) as representative New World monkeys [46]. The sequence (MH177805) of human *cox1* was used as an out-group. Finally, the Hasegawa-Kishino-Yano (*+G, +I*) [87] was selected as a best fit model according to the Akaike Information Criterion (AIC) option in MEGA6 [88]. The maximum likelihood (ML) phylogenetic inference was used with 1000 bootstrap replicates to generate the phylogenetic tree using the same software.

### 4.4. Molecular Screening for Filaria and Wolbachia

First, all blood samples were screened for the presence of filaria and *Wolbachia* DNAs using, respectively, the pan-filarial [Pan-fil 28S] and pan-*Wolbachia* [All-Wol 16S] qPCRs, as described elsewhere [24].

### 4.5. Molecular Characterization of Filariids and their Associated Wolbachia Using Generic Primers

Samples positive for filaria and *Wolbachia* by qPCR were subjected to amplification and sequencing analysis using the pan-Nematoda-*18S* primers [61] and pan-filarial *cox1* based PCR [Pan-fil *cox*1] [24] to generate 1127–1155 bp and 509 bp from the filarial *18S* and *cox1* genes, respectively. The third PCR system [W16S-Spec] PCR [89] was used to amplify 438 bp from the *16S* gene of *Wolbachia* spp. (Table 3).

### 4.6. Molecular Characterization of Filariids Using Genus Specific PCR Assays

#### 4.6.1. Design of Oligonucleotides

In order to complete the molecular characterization of filariids detected by the *18S* and *cox1* genes, we targeted the Internal Transcribed Spacer 1 (*ITS1*) gene to design genus-specific PCR assays targeting *Brugia* and *Mansonella* species. The choice for this gene was based on the following criteria: a higher divergence between filarial species especially among *Brugia* species [90], its tandem repeat that increases PCR sensitivity [91] and its availability in the GenBank database for these species. Three PCR assays were designed by the alignment of *ITS1* sequences of *Brugia* sp. (HE856316), *B. malayi* (EU419346, JQ327149), *B. timori* (AF499132), *B. pahangi* (EU373628), *M. ozzardi* (MN432519, LT623912, AF228559), *M. perstans* (MN432520, KJ631373, EU272184) and *M. mariae* (KX932484) against 33 sequences (data not showed) from a representative member of Onchocercidae using the MUSCLE application within DNAstar software [92]. Three genus specific PCR systems were proposed (Table 3). This includes two PCRs: one specific for *Brugia* spp. [Brug-gen-spec] and the other specific for *Mansonella* spp. [Manso-gen-spec], and qPCR system [Brug-gen-spec qPCR] targeting *Brugia* spp.

Assay specificity was confirmed in silico and in vitro for each system, as described elsewhere [24]. Briefly, the in silico validation was conducted using Primer-BLAST [93]. Genomic DNA of *M. perstens* was used to validate the PCR for *Mansonella*, while the *B. malayi* DNA was used to validate both the qPCR and PCR for *Brugia* spp. Moreover, all PCR assays were challenged against the genomic DNA of filariids other than *Brugia* and *Mansonella,* as well as several nematodes, arthropods, vertebrate hosts (e.g., human, monkey, donkey, horse, cattle, mouse and dog) and laboratory-maintained colonies [24].

#### 4.6.2. Amplification, Sequencing and Run Protocol

All blood samples from howler monkeys were screened for the presence of *Mansonella* and *Brugia* DNA using the genus specific PCR. The PCR reactions were carried out in a total volume of 50 µL, comprising 25 µL of AmpliTaq Gold master mix (Thermo Fisher Scientific, Saint Herblain, France), 18 µL of ultrapure water free of DNAse-RNAse, 1 µL of each primer and 5 µL of genomic DNA. PCR reactions were run under the following protocol: the incubation step at 95 °C for 15 min, 40 cycles of one minute at 95 °C, 30 s for the annealing at a different melting temperature for each PCR assays (Table 3), and 72 °C of elongation step (Table 3) with a final extension step of five minutes at 72 °C. PCR reactions were performed in a Peltier PTC-200 model thermal cycler (MJ Research Inc., Watertown, MA, USA).

DNA amplicons generated throughout each PCR reaction were purified using NucleoFast^®^ 96 PCR DNA purification plate (Macherey Nagel EURL, Hoerdt, France). Purified DNAs were subjected to the second amplification using the BigDye™ Terminator v3.1 Cycle Sequencing Kit (Perkin Elmer Applied Biosystems, Foster City, CA, USA), then the BigDye PCR products were purified on the Sephadex G-50 Superfine gel filtration resin prior to sequencing on the ABI Prism 3130XL (Applied Biosystems, Courtaboeuf, France).

#### 4.6.3. Molecular Screening for Brugia

In order to reveal the infection rate of *Brugia* spp., all the samples were subjected to the amplification using the genus-specific qPCR. The qPCR reaction was performed in a total volume of 20 µL including 5 μL of DNA template, 10 μL of Master Mix Roche (Eurogentec France, Angers, France), 3 µL of ultra-purified water DNAse-RNAse free and 0.5 µL of each primer, UDG and each probe. The TaqMan reaction of both systems was run using the same cycling conditions. This included two hold steps at 50 °C and 95 °C for 2 and 15 min, respectively, followed by 40 cycles of two steps each (f 95 °C for 30 s and 60 °C for 30 s). The qPCR reaction was performed in a CFX96 Real-Time system (Bio-Rad Laboratories, Foster City, CA, USA).

### 4.7. Phylogenetic Analysis

First, nucleotide sequences of the filarial *cox1*, *18S* and *ITS1* genes, as well as the *16S* gene of *Wolbachia,* were assembled and edited by Chromas-Pro 2.0.0 (http://technelysium.com.au/wp/chromaspro/). The absence of co-amplification of nuclear mitochondrial genes (numts) was verified by aligning the obtained *cox1* sequences with the *Mansonella ozzardi* mitogenome (KX822021) [45]. Furthermore, ambiguities in the sequence chromatograms, stop codons and indels were visually verified, as recommended in Reference [94]. All the sequences were subjected separately to a preliminary analysis using Basic Local Alignment Search Tool (BLAST) [95].

Both the nuclear *18S* rRNA alone or concatenated with the *ITS1* (if amplified) gene from each filarial species generated through the present study were separately aligned against the previously published sequences from the complete rRNA sequences or draft/complete genomes from the Onchocercidae clade ONC2, ONC3, ONC4 and ONC5 [56]. While, the *cox1* sequences were aligned against the representative members of the clade ONC4 and ONC5 encountered in primates [56]. The *Wolbachia 16S* DNA sequences were aligned against the representative members of *Wolbachia* lineages (C, D, F and J) infecting filarial parasites [11,16]. MAFFT alignment was performed on the concatenated nuclear (*18S* rRNA and *ITS1*) sequences using DNAstar software [92], while the *18S*, the *cox1* and the *16S* DNA sequences were aligned using ClustalW application within Bioedit v.7.2.5. [96]. The Akaike Information Criterion (AIC) option in MEGA6 [88] was used to establish the best nucleotide substitution model adapted to each sequence alignment. The Kimura 2-parameter model (*+G*) [97] was used to generate the *18S* and the *16S* trees, while the Tamura 3-parameter model (*+I*) [98] and the General Time Reversible model (*+G*, *+I*) [98] were, respectively, used for the concatenated rRNA (*18S* and *ITS1*) and the *cox1* alignments. A maximum likelihood (ML) phylogenetic inference was used with 1000 bootstrap replicates to generate the phylogenetic tree in MEGA6 [88]. *Gongylonema nepalensis* (LC278392) rRNA sequence, both Filarioidea species (KP728088) and *Physaloptera amazonica* (MK309356) *cox1* sequences and the *16S* DNA sequence of *Rickettsia* sp. (AB795333) were used as out groups to root the trees.

In addition, we generated another *cox1* alignment, including the representative members of all the Onchocercidae clades (ONC1, ONC2, ONC3, ONC4 and ONC5) [56]. Two Filariidae and four Physalopteridae sequences were included as out-groups. The interspecific nucleotide pairwise distance (IND) was used to estimate the evolutionary divergence between *cox1* sequences among Onchocercidae. Standard error was obtained by a bootstrap procedure with 1000 replicates. Analyses were inferred on MEGA6 software [88], based on the Maximum Composite Likelihood model [99]. A scatter chart based on the IND between Onchocercidae members and the *cox1* sequences generated in the present study was drowned using XLSTAT Addinsoft version 4.1 (XLSTAT 2019: Data Analysis and Statistical Solution for Microsoft Excel, Paris, France).

Finally, COI protein sequences of *Brugia* species (Protein Id: QIL51350, QDE55703, ALR73830, QDE55700 and ALR73832) and those of *Mansonella* species (Protein Id: CAO83087, QHA95050, AVA30206, CAO83074 and SCW25063) were retrieved from the GenBank database and aligned against the COI sequences obtained from monkeys B9 and B8, respectively. The alignment was performed using the ClustalW application within Bioedit v.7.2.5. [96]. Amino acids conservation between the COI sequences from this study comparatively to GenBank sequences was visualized on the CLC Sequence Viewer 7 (CLC Bio Qiagen, Aarhus, Denmark).

## 5. Conclusions

In this study, we phylogenetically describe filarial parasites belonging to three distinct genera: *Mansonella* sp. *Brugia* sp. and an unidentified Onchocercidae species. Funding extends the presence of *Brugia* sp. and the unidentified Onchocercidae species to Guiana monkeys. In addition, phylogenetic analyses highlight the necessity of completing the classification of filariasis of neo-tropical monkeys by combining morphological and molecular-based identification for an integrative taxonomical perspective. Filaria associated *Wolbachia* can be used as diagnostic markers since they are genus specific endosymbionts. Regarding the presence of *Brugia* sp. in Guiana monkeys, the same genotype circulates in French Guiana dogs, suggesting host diversity of this filariids. We therefore developed a novel qPCR assay that could be useful for the surveillance of brugian filariasis in vectors, animals, and humans. Further studies will be needed to shed light on the life cycle, epidemiology and circulation of this potentially zoonotic parasite.

## Figures and Tables

**Table 1:** Results of molecular assays used for the identification of filariids and their associated *Wolbachia* in the blood of red howler monkeys from French Guiana.

Sample Code	Filarial DNA						*Wolbachia* DNA		Decision
	Filariids			ITS genus-specific PCRs			*Wolbachia* 16S-specific PCRs		
	28S qPCR	*18S* PCR	*COI* PCR	*Mansonella* spp. *PCR*	*Brugia* spp. PCR	*Brugia* spp. qPCR	*Wolbachia 16S* qPCR	*Wolbachia 16S* PCR	Combined Assays
B1	N/A	N/A	N/A	N/A	N/A	Neg.	Neg.	N/A	Negative.
B2	Pos.	*Mansonella* sp. [MT336169]	O/P	*Mansonella* sp. [MT341515]	N/A	Pos.	Neg.	N/A	*Mansonella* sp. + *Brugia* sp.
B3	Pos.	*Mansonella* sp. [MT336170]	O/P	*Mansonella* sp. [MT341516]	*Brugia* sp. [MT341511]	Pos.	Pos.	O/P	*Mansonella* sp. + *Brugia* sp.
B4	Pos.	*Mansonella* sp. [MT336171]	O/P	*Mansonella* sp. [MT341517]	*Brugia* sp. [MT341512]	Pos.	Pos.	*W-Mansonella sp.* [MT231961]	*Mansonella* sp. + *Brugia* sp.
B5	Pos.	unidentified Onchocercidae species [MT336175]	O/P	*Mansonella* sp. [MT341518]	N/A	Neg.	Pos.	*W-Mansonella sp.* [MT231962]	*Mansonella* sp. + unidentified Onchocercidae species
B6	Pos.	*Mansonella* sp. [MT336172]	O/P	*Mansonella* sp. [MT341519]	N/A	Neg.	Pos.	*W-Brugia* sp. [MT231964]	*Mansonella* sp. + *Brugia* sp.
B7	Pos.	*Mansonella* sp. [MT336173]	O/P	*Mansonella* sp. [MT341520]	*Brugia* sp. [MT341513]	Pos.	Neg.	N/A	*Mansonella* sp. + *Brugia* sp.
B8	Pos.	*Mansonella* sp. [MT336174]	*Mansonella* sp. [MT724663]	*Mansonella* sp. [MT341521]	N/A	Neg.	Pos.	*W-Mansonella sp.* [MT231963]	*Mansonella* sp.
B9	Pos.	*Brugia* sp. [MT336168]	*Brugia* sp. [MT724693]	N/A	*Brugia* sp. [MT341514]	Pos.	Pos.	*W-Brugia* sp. [MT231965]	*Brugia* sp.

N/A: no amplification, O/P: overlapping peaks on the electropherograms, Pos: positive reaction, Neg: negative reaction, *W-Mansonella* sp.: *Wolbachia* endosymbiont of *Mansonella* sp., *W-Brugia* sp.: *Wolbachia* endosymbiont of *Brugia* sp. GenBank accession numbers are given in square brackets.

**Table 2:** Filarial parasites and host diversity from neotropic monkeys.

Genera	Species	Host	References
*Mansonella* (Faust, 1929), *Mansonella (Tetrapetalonema)* comb. n. (Faust 1935)	*Mansonella (T.) marmosetae* (Faust 1935)	*Saguinus geoffroyi*, *Saimiri oerstedii oerstedii*, *Ateles paniscus*, *Saimiri boliviensis*, *Saimiri sciureus* and *Alouatta* spp.	[44,66,67,76]
	*Mansonella (T.) zakii* (Nagaty 1935)	*Leontopithecus (= Leontocebus) rosalia*	
	*Mansonella (T.) panamensis* (McCoy 1936)	*Cebus capucinus*, *Saimiri oerstedii oerstedii*, *Aotus lemurinus zonalis*, *C. apella* and *A. trivirgatus*	
	*Mansonella (T.) atelensis atelensis* (McCoy 1935)	*Ateles geoffroyi*, *A. fusciceps rufiventris*	
	*Mansonella (T.) atelensis amazonae* (Bain and Guerrero 2015)	*Cebus olivaceus*	
	*Mansonella (T.) parvum* (McCoy 1936)	*Cebus capucinus*, *Saimiri oerstedii oerstedii*	
	*Mansonella (T.) obtusa* (McCoy 1936)	*Cebus capucinus*, *C. capucinus*, *C. albifrons*, *Saimiri oerstedii oerstedii*	
	*Mansonella (T.) tamarinae* (Dunn and Lambrecht 1963)	*Saguinus (= Tamarinus) nigricollis*	
	*Mansonella (T.) barbascalensis* (Esslinger and Gardiner 1974)	*Aotus trivirgatus*	
	*Mansonella (T.) mystaxi* (Eberhard 1978)	*Saguinus mystax mystax*	
	*Mansonella (T.) saimiri* (Esslinger 1981)	*Saimiri sciureus*	
	*Mansonella (T.) peruviana* (Bain, Petit and Rosales-Loesener 1986)	*Saimiri sciureus*	
	*Mansonella (T.) colombiensis* (Esslinger 1982)	*Saimiri sciureus*, *Cebus apella*	
	*Mansonella (T.) mariae* (Petit, Bain and Roussilhon 1985)	*Saimiri sciureus*	
*Dipetalonema* (Diesing 1861)	***D. gracile* (Rudolphi 1819)**	*Saimiri sciureus*, *Cebus albifrons*, *A. geoffroyi*, *Aotus lemurinus*, *Ateles chamek*, *Ateles fusciceps*, *Ateles geoffroyi*, *Ateles paniscus*, *Cebus apella*, *Cebus capucinus*, *Cebus* spp., *Lagothrix lagothricha*, *Saguinus mystax*, *Saguinus nigricollis*, *Saimiri oerstedii*, *Saimiri sciureus*, *Saimiri sciureus*, *Sapajus macrocephalus*, *B. arachnoïdes*, *L. rosalia*, *Leontopithecus chrysopygus*, *Saguinus bicolor*, *Cebus albifrons*	[76,77,78,79,80,81,82]
	***D. graciliformis* (Freitas 1964)**	*Saguinus midas*	
	*D. robini* (Petit et al. 1985)	*Saimiri sciureus*, *Sapajus nigritus*, *Saimiri boliviensis*, *Cebus* spp.	
	*D. freitasi* (Bain, Diagne and Muller 1987)	*Cebus capucinus*	
	***D. caudispina* (Molin 1858)**	*Alouatta seniculus*, *Ateles paniscus*, *Brachyteles arachnoides*, *Cebus albifrons*, *Cebus apella*, *Lagothrix lagotricha*, *Leontopithecus rosalia*, *Saimiri sciureus*, *Saimiri sciureus*, *Sapajus macrocephalus*	
	*D. obtusa* (McCoy 1936)	*Cebus albifron*, *Cebus capucinus*	
	*D. yatesi* (Julians 2007)	*Ateles chamek*	

Species in bold are occurring in French Guiana monkeys.

**Table 3:** The primers and probes used in this study.

System Name	Target Gene	Primer and Probe Name	Sequence (5′–3′)	Amplicon Size (bp)	Tm/Elongation Time	Assay Specificity	Ref.
Pan-fil 28S qPCR-based system	*LSU* rRNA *(28S)*	qFil-28S-F	TTGTTTGAGATTGCAGCCCA	151	60 °C/30”	Filariids	[24]
		qFil-28S-P	6FAM-CAAGTACCGTGAGGGAAAGT-TAMRA				
		qFil-28S-R	GTTTCCATCTCAGCGGTTTC				
All-Wol 16S qPCR-based system	*16S* rRNA gene	all.Wol.16S.301-F	TGGAACTGAGATACGGTCCAG	177	61 °C/30”	*Wolbachia*	
		all.Wol.16S.347-P	6FAM-AATATTGGACAATGGGCGAA-TAMRA				
		all.Wol.16S.478-R	GCACGGAGTTAGCCAGGACT				
16S W-Spec		W-Specf	CATACC TATTCGAAGGGATAG	438	60 °C/1’		[89]
		W-Specr	AGCTTCGAGTGAA ACCAATTC				
Brug-gen-spec qPCR	Internal Transcribed Spacer 1 (*ITS1*)	Brug.ITS.f.260	AGCGATAGCTTAATTAATTTTACCATTT	161	61 °C/30”	*Brugia* spp.	This study
		Brug.ITS.p.307	6FAM- GCATTTATGCTAGATATGCTACCAA-TAMRA				
		Brug.ITS.r.421	CCACCGCTAAGAGTTAAAAAAATT				
Brug-gen-spec PCR		Fil.ITS.f:	GAACCTGCGGAAGGATCA	417–441	54 °C/30”		
		Brug.ITS.r	CCACCGCTAAGAGTTAAAAAAATT				
Manso-gen-spec PCR		Fil.ITS.f:	GAACCTGCGGAAGGATCA	333–345	55 °C/30”	*Mansonella* spp.	
		Manso.ITS.r	TGTGTATTTATTTGTTGGTAGCATATT				
	*SSU* rRNA *(18S)*	Fwd.18S.631	TCGTCATTGCTGCGGTTAAA	1127–1155	54 °C/1’30”	Nematoda	[61]
		Rwd.18S.1825r	GGTTCAAGCCACTGCGATTAA				
Pan-fil *cox1* PCR	Cytochrome c oxidase subunit 1 gene (*cox1*)	Fwd.957	ATRGTTTATCAGTCTTTTTTTATTGG	509	52 °C/1’	Filariids	[24]
		Rwd.1465	GCAATYCAAATAGAAGCAAAAGT				
dg-Folmer’s primers		dgLCO-1490	GGTCAACAAATCATAAAGAYATYGG	708	44 °C/40”	Metazoans	[86]
		dgHCO-2198	TAAACTTCAGGGTGACCAAARAAYCA				

## Supplementary Materials

The following are available online at https://www.mdpi.com/2076-0817/9/8/626/s1, Figure S1: *18S* sequences alignment showing the nucleotide conservation of the unidentified Onchocercidae species obtained from howler monkey against the GenBank sequences of *O. volvulus* and *L. loa*, Table S1: Estimates of the evolutionary divergence between the cytochrome c oxidase subunit I (*cox1*) sequences of *Mansonella* sp. and *Brugia* sp. obtained in this study comparatively with Onchocercidae members from GenBank database.
