# Supplementary figures and images for: New Molecular Data on Filaria and its Wolbachia from Red Howler Monkeys (Alouatta macconnelli) in French Guiana—A Preliminary Study
Source: Pathogens. 2020 Jul 31;9(8):626. doi: 10.3390/pathogens9080626 (PMC7460519; doi:10.3390/pathogens9080626)

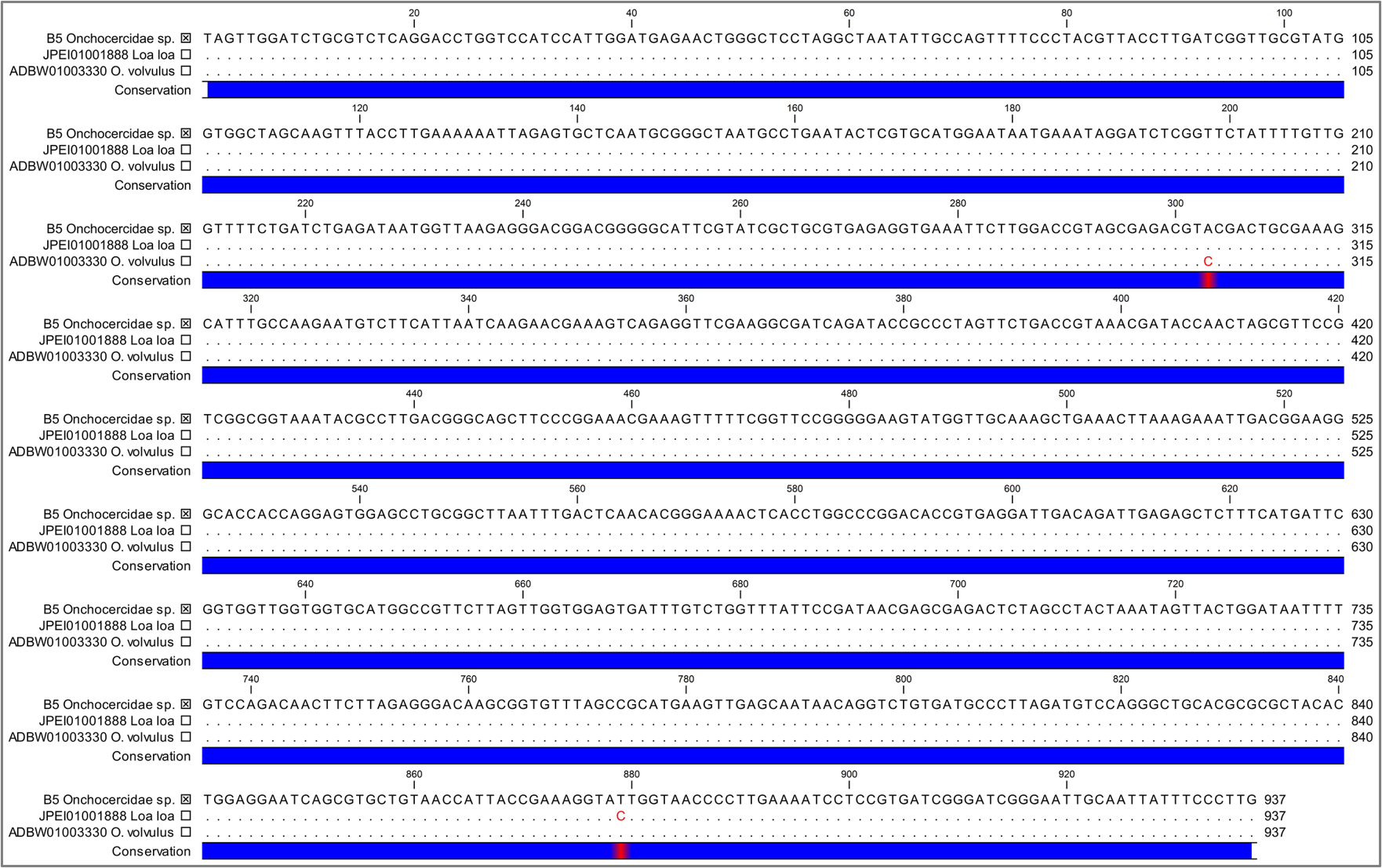

Supplement: Supplementary file 1 [file pathogens-09-00626-s001.zip › Figure S1.tif]
